# Prognostic Value of Long Non-Coding RNA HULC and MALAT1 Following the Curative Resection of Hepatocellular Carcinoma

**DOI:** 10.1038/s41598-017-16260-1

**Published:** 2017-11-23

**Authors:** Fuminori Sonohara, Yoshikuni Inokawa, Masamichi Hayashi, Suguru Yamada, Hiroyuki Sugimoto, Tsutomu Fujii, Yasuhiro Kodera, Shuji Nomoto

**Affiliations:** 1 Department of Gastroenterological Surgery, Nagoya University Graduate School of Medicine, Nagoya, Japan; 2 Department of Surgery, Aichi-Gakuin University School of Dentistry, Nagoya, Japan

## Abstract

Long non-coding RNAs (lncRNAs) were shown to be the crucial regulators of the many diseases. In this study, the expressions of lncRNAs were investigated in resected 158 hepatocellular carcinomas (HCCs) to evaluate the effects of their expression levels on prognosis. The expression levels of *HULC* and *MALAT1* were shown to be significantly higher in the normal background tissue of HCC than those in the normal liver tissue of metastatic liver tumor without hepatitis (*HULC*: fold change 14.9, *P* = 1.7e-06; *MALAT1*: fold change 17.5, *P* = 1.2e-06. The formation of capsule was shown to be correlated with the increased expression of *HULC* (*P* = 0.041), while the size of HCC under 2 cm was correlated with a decrease in *MALAT1* expression (*P* = 0.019). The levels of serum alpha-fetoprotein above 20 ng/mL indicated a decreased expression of both *HULC* and *MALAT1* (*HULC*: *P* = 0.017; *MALAT1*: *P* = 0.0036). The increase in the expression levels of *MALAT1* in HCC tissues was significantly correlated with better overall survival (*HULC*: *P* = 0.099, *MALAT1*: *P* = 0.028). Thus, the expression of these lncRNAs in HCC potentially correlates with the HCC malignancy and they represent potential prognostic biomarkers of the resected HCC.

## Introduction

Hepatocellular carcinoma (HCC) represents the fifth most common malignancy and the third most common cause of cancer-related death worldwide^1^. Hepatic resection is one of the most effective treatments for non-metastatic HCC^2–4^. However, after the curative resection, ~80% of patients develop intrahepatic recurrence, and 50% die within 5 years^5^. Therefore, although the surgical resection of early HCC can be curative, the pronounced tendency toward recurrence represents a major concern^6^.

Intrahepatic HCC recurrence is categorized as intrahepatic metastasis (IM) or multicentric occurrence (MO). IM refers to the HCC foci developing from tumor cells that spread into the remnant liver via the portal vein before or during hepatic resection. MO refers to the development of postsurgical HCC foci due to chronic active hepatitis or cirrhosis, caused by viruses, alcohol, toxins, or other HCC-relevant risk factors^7–10^. The results of previous studies indicated that the clinical progression and outcome of IM and MO differ significantly^11^. Considering these specific characteristics of recurrence, focusing on the analysis of tumor tissue alone may be insufficient. In all HCC cases, the consideration of any correlations between the HCC tissue and the surrounding non-tumor tissue is important^12^. We demonstrated previously that the alterations in the non-malignant liver tissue gene profiles can be used for the prognostic purposes in HCC^13^.

Long non-coding RNAs (lncRNAs) were identified as the key players in tumorigenesis and tumor progression. Many reports showed that lncRNA dysregulation is linked to the development of many diseases, including cancer^14–16^. Therefore, we hypothesized that the expression of lncRNAs in the background liver tissue of HCC may be important for HCC prognosis as well. We attempted to identify novel lncRNAs related to HCC prognosis by using the microarray analysis of their expression in the background liver tissue of HCC patients and normal liver tissue without HCC and/or hepatitis. We evaluated the differences in lncRNA expression levels in HCC and corresponding non-tumor tissues, and identified the unique prognostic markers for the HCC.

## Methods

### Patients and Samples

For microarray analysis, the representative non-tumor liver tissue of a HCC patient (CN, for the corresponding normal) was obtained from a typical HCC patient, a 58-year-old man during hepatectomy. This HCC was caused by the chronic hepatitis C infection, and it recurred three years after the resection. Pathology confirmed the absence of cancerous regions from the CN sample. As controls, non-cancerous liver tissue without hepatitis (SN, for super normal) was obtained from 11 patients with liver metastases who underwent hepatectomy at Nagoya University Hospital, Japan. Their primary diseases were colorectal cancer (n = 5), gastrointestinal stromal tumor (n = 2), or gastric cancer, esophageal cancer, cervical cancer, or tongue cancer (n = 1, each).

For real-time quantitative reverse transcription PCR (RT-qPCR), HCC and CN tissue samples were collected from 158 consecutive patients who underwent curative resection at Nagoya University Hospital between January 1998 and December 2011. Resection was defined as curative when all gross tumors were removed completely; incidentally found small lesions suspected to be HCC that were treated intraoperatively by radiofrequency therapy or microwave coagulation therapy were also regarded as curative cases. Patient characteristics are summarized in Table 1. After surgery, all patients were monitored by performing blood examinations, ultrasonography, and computed tomography. In the cases of possible recurrence, angiography was performed in order to confirm it. The median follow-up duration of all cases was 48.5 months (range, 0.3 to 193.8 months). All tumor tissue samples were histologically confirmed as HCC samples by pathologists. All surgically obtained tissue samples were immediately frozen in the liquid nitrogen and stored at −80 °C until further analyses. This study and all procedures were approved by the Institutional Review Board at Nagoya University and all patients provided written informed consent. All clinical investigations were conducted in accordance with the principles of the Declaration of Helsinki.

**Table 1:** HCC patient characteristics (n = 158).

Characteristics		Value
Age (years)	Median (range)	65 (37–84)
Sex	Male: female	132 (84):26 (16)
Viral infection	HBV:HCV:non-HBV/HCV	41 (26):92 (58):28 (18)
Child-Pugh classification	A:B	148 (94):9 (6)
Liver damage classification	A:B:C	126 (83):25 (16):1 (1)
Albumin (g/dL)	Median (range)	3.9 (2.3–4.9)
Total bilirubin (mg/dL)	Median (range)	0.7 (0.2–7.3)
PT (%)	Median (range)	89.7 (46.9–138)
AFP (ng/mL)	Median (range)	17 (0.8–119923)
Tumor size (cm)	Median (range)	3.5 (0.15–15)
Tumor multiplicity	Solitary: multiple	124 (78): 34 (22)
ICG-R15 (%)	Median (range)	11.5 (1.6–35.2)
Stage	I:II:III:IV	17 (11):82 (52):40 (26):17 (11)

Abbreviations: HCC, hepatocellular carcinoma; n, number; HBV, hepatitis B virus; HCV, hepatitis C virus; PT, prothrombin time; AFP, alpha-fetoprotein; ICG-R15, retention rate of indocyanine green 15 minutes after administration.

### Gene Expression Analysis

#### Microarray

Total RNA was extracted from the fresh-frozen CN and SN tissue samples using a Qiagen miRNeasy mini-kit (Qiagen, Hilden, Germany). Eleven SN samples were pooled so that the individual differences are eliminated. RNA quality was confirmed based on the RNA integrity number ≥8 as measured using an Agilent 2100 Bioanalyzer (Agilent, Santa Clara, CA, USA). RNA was labeled with cyanine-3 dye using a Quick Amp labeling kit (Agilent, Santa Clara, CA, USA) and hybridized to Agilent whole human genome (4 × 44 K) microarrays for 17 h in a rotating SciGene model 700 oven (SciGene, Sunnyvale, CA, USA). The arrays were scanned (Agilent DNA microarray scanner), and the data were feature-extracted using Feature Extraction software 10.5.1.1 and statistically analyzed using the default settings for GeneSpring GX 11.0.1 software (Agilent, Santa Clara, CA, USA)^17^.

#### RT-qPCR

Total cDNA was developed from the RNA extracted from each fresh-frozen tissue with M-MLV Reverse Transcriptase (Invitrogen, Carlsbad, CA, USA). This total cDNA was used as a template for the next step of quantitative PCR. PCR was performed using SYBR Premix Ex Taq II (Takara Clontech, Kyoto, Japan) under the following conditions: denaturing at 95 °C for 10 s, 40 cycles of denaturing at 95 °C for 5 s, and annealing/extension at 60 °C for 30 s. The SYBR Green signal was detected in real-time using StepOne Plus Real-Time PCR System (Life Technologies, Carlsbad, CA, USA). The relative quantification method was used and each gene expression level was determined to be the expression level of the target gene, glyceraldehyde-3-phosphate dehydrogenase (*GAPDH)*, for each sample.

The PCR primers used in current study were for the 84 base pairs fragment of Hepatocellular Carcinoma Up-Regulated Long Non-Coding RNA (*HULC*) (sense, 5′-ACTCTGAAGTAAAGGCCGGAA-3′; antisense 5′-GCCAGGAAACTTCTTGCTTGA-3′) and for the 14 base pairs fragment of Metastasis Associated Lung Adenocarcinoma Transcript 1 (*MALAT1*) (sense, 5′-CCAGTTGAATTCACCAGTGGAC-3′; antisense 5′ - AGTTTGCTCACATGCCAGTTAC-3′). *GAPDH* (sense, 5′-GAAGGTGAAGGTCGGAGTC-3′; antisense 5′-GAAGATGGTGATGGGATTTC-3′; probe 5′-[Fl] CAAGCTTCCCGTTCTCAGCC [Fl-Q]-3′) expression was quantified in each sample in order to perform the normalization. All RT-qPCR experiments were performed in triplicate, including the template-omitted negative controls.

### Public Available Dataset

Normalized TCGA RNA-sequencing data for hepatocellular carcinoma was downloaded from the Broad GDAC Firehose (http://gdac.broadinstitute.org/, accessed on November 1st, 2017). This dataset includes 360 HCC cases including seven HCCs mixed with hepatocholangiocarcinoma and two cases with fibrolamellar carcinoma. There are 266 cases with information of recurrence-free survival (RFS) and 336 cases with information of overall survival (OS) out of the 360 cases.

### Statistical Analyses

Continuous variables were expressed as median (range) and expression of each target gene was compared by a Wilcoxon signed-rank test. Categorical variables were compared using the χ^2^ or Fisher’s exact tests, as appropriate. Correlation between each gene expression was analyzed with Spearman’s rank correlation coefficient. OS and RFS rate at each point of the follow-up time were estimated using the Kaplan-Meier method and compared using a log-rank test. The Cox proportional hazard regression model was used to perform univariate analysis for OS. All statistical analyses were performed using R (version 3.4.1; http//www.r-project.org/) and statistical significance was set at *P* < 0.05, which was obtained using two-tailed tests.

## Results

### Expression Profiling

To identify novel tumor-related lncRNA in HCC, we compared the non-coding RNA expression profiles of a CN sample and the pooled SN samples. Microarray analysis revealed that lncRNAs upregulated in the CN sample (fold change higher than 1.2) were Urothelial Cancer Associated 1 (*UCA1*: fold change 7.98), *HULC* (fold change 3.49), *MALAT1* (fold change 1.85), Growth Arrest Specific 5 (*GAS5*: fold change 1.28), and Taurine Up-Regulated 1 (*TUG1*: fold change 1.27) (Supplementary Table S1).

### SN, CN, and HCC Tissue Sample Analysis

**Figure 1:**
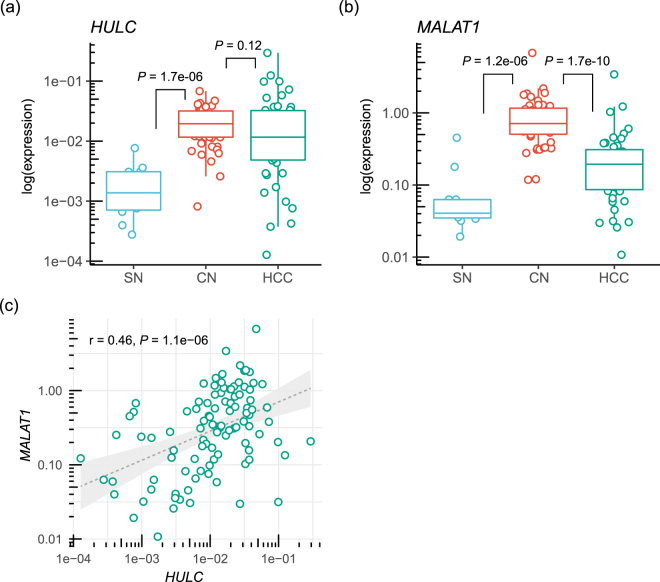
*HULC* and *MALAT1* expression levels in SN, CN, and HCC samples. (**a**) *HULC* expression levels in SN, CN, and HCC samples. (**b**) *MALAT1* expression in SN, CN, and HCC samples. (**c**) Correlation between *HULC* expression and *MALAT1* expression in HCC. CN, corresponding normal; HCC, hepatocellular carcinoma; HULC, Hepatocellular Carcinoma Up-Regulated Long Non-Coding RNA; MALAT1, Metastasis Associated Lung Adenocarcinoma Transcript 1.

As determined by analyzing 11 SN and 48 paired CN and HCC, *HULC* and *MALAT1* expression levels were significantly higher in the CN tissue samples (*HULC*: median, 0.019 [range, 0.0008 to 0.068]; *MALAT1*: 0.71 [0.12 to 6.8]; n = 48) than in the SN tissue samples (*HULC*: 0.0013 [0.0002 to 0.0076], *P* = 1.7e-06; *MALAT1*: 0.041 [0.019 to 0.45], *P* = 1.2e-06; n = 11) (Fig. 1a and b). However, no significant difference in *HULC* expression between CN and HCC was observed (0.012 [range, 0.0001 to 0.29], *P* = 0.12, n = 158) (Fig. 1a). *MALAT1* expression levels in the HCC samples (0.19 [range, 0.011 to 3.4], n = 158) were shown to be significantly lower than those in the CN samples (*P* = 1.7e-10) (Fig. 1b). There was a weak positive correlation between HULC and MALAT1 expression in HCC tissue (correlation coefficient r = 0.46, *P* = 1.1e-06) (Fig. 1c).

**Figure 2:**
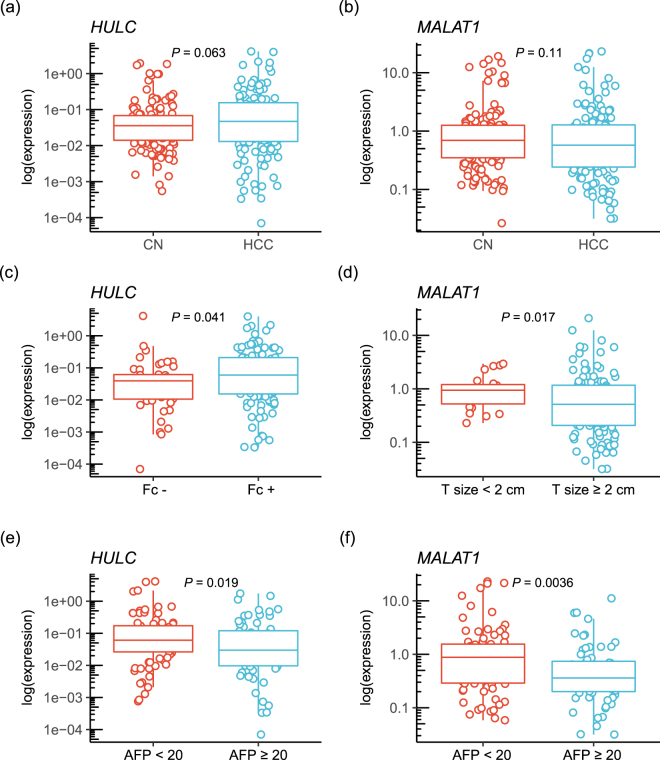
*HULC* and *MALAT1* expression levels in 158 HCC samples. (**a**) *HULC* expression in CN and HCC samples. (**b**) *MALAT1* expression levels in CN and HCC tissue samples. (**c**) *HULC* expression in the HCC cases accompanied by the formation of capsule (Fc) or without Fc. (**d**) *MALAT1* expression in HCC samples larger or smaller than 2 cm. (**e**) *HULC* expression level in the HCC cases with higher serum AFP levels. (**f**) *MALAT1* expression level in the HCC cases with higher serum AFP levels. CN, corresponding normal; HCC, hepatocellular carcinoma; Fc, formation of capsule; AFP, alpha-fetoprotein; HULC, Hepatocellular Carcinoma Up-Regulated Long Non-Coding RNA; MALAT1, Metastasis Associated Lung Adenocarcinoma Transcript 1.

When 158 curably resected HCC tissue samples were analyzed, *HULC* expression levels were shown to be higher in the HCC samples (0.047 [range, 0.00007 to 4.1], n = 158) in comparison with those in the CN tissues (0.036 [0.0005 to 1.9], *P* = 0.063; n = 58) (Fig. 2a). However, the expression of *MALAT1* did not differ between CN (0.69 [0.026 to 19.1], n = 158) and HCC tissues (0.57 [0.032 to 23.1], *P* = 0.11; n = 158) (Fig. 2b). In the CN samples, no significant difference was found between other clinicopathological factors and *HULC* or *MALAT1* expression. However, *HULC* expression levels in HCC cases where the capsular formation can be observed (0.059 [0.0003 to 4.0], n = 101) was significantly higher than in the HCC samples without capsular formation (0.039 [0.00007 to 4.1], *P* = 0.041, n = 45) (Fig. 2c). *MALAT1* expression levels in HCC samples larger than 2 cm (0.51 [0.031 to 21], n = 116) were significantly lower than those in the HCC under 2 cm (0.94 [0.23 to 2.9], *P* = 0.019, n = 23) (Fig. 2d). The expression of *HULC* and *MALAT1* in HCC cases with higher serum alpha-fetoprotein (AFP) levels (>20 ng/mL) (*HULC*: 0.030 [0.00007 to 1.7], n = 64; *MALAT1*: 0.36 [0.031 to 11.1, n = 64) were significantly lower than those in the HCC samples with lower serum AFP values (<20 ng/mL) (*HULC*: 0.061 [0.0007 to 4.1], *P* = 0.019, n = 80; *MALAT1*: 0.88 [0.058 to 23.1], *P* = 0.0036, n = 81) (Fig. 2e and f).

### HULC and MALAT1 Correlation with HCC Prognosis

**Figure 3:**
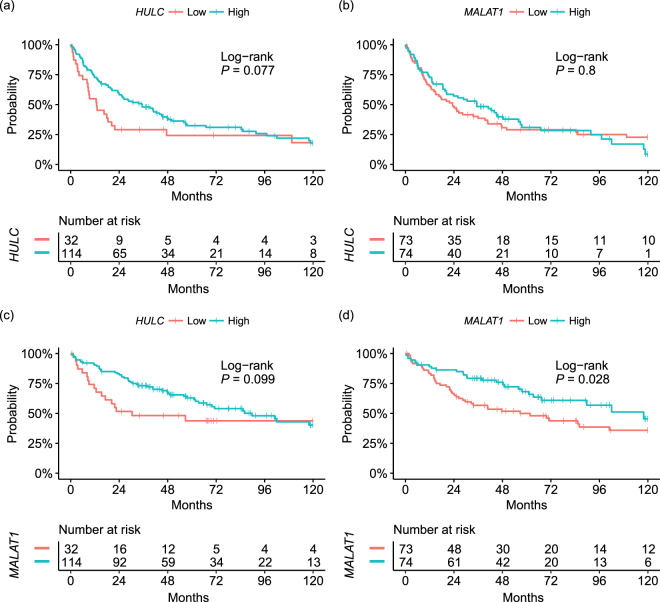
Survival analysis stratified by *HULC* and *MALAT1* expression levels in HCC tissues. (**a**) RFS analysis stratified by *HULC* expression. (**b**) OS analysis stratified by *HULC* expression. (**c**) RFS analysis stratified by *MALAT1* expression. (**d**) OS analysis stratified by *MALAT1* expression. HULC, Hepatocellular Carcinoma Up-Regulated Long Non-Coding RNA; MALAT1, Metastasis Associated Lung Adenocarcinoma Transcript 1; OS, overall survival; RFS, recurrence-free survival.

Based on the results obtained by the real time RT-qPCR, 158 HCC cases were divided into two groups, according to the *HULC* and *MALAT1* expression levels in HCC tissues (*HULC*: < 0.01 and ≥ 0.01; *MALAT1*: < median and ≥ median) and the effects of the expression levels on RFS and OS were evaluated. The distribution of each clinicopathological features stratified by *HULC* and *MALAT1* expression levels in HCC is shown in Supplementary Table S2 and S3 (online). OS and RFS analysis stratified by *HULC* expression in HCC revealed high expression of *HULC* tended to have better RFS and OS but there was no significance between cases with high *HULC* and low *HULC* (RFS: *P* = 0.077; OS: *P* = 0.099) (Fig. 3a and b). In HCC tissue samples, no significant difference in RFS linked to the *MALAT1* expression level was observed (*P* = 0.8) (Fig. 3c). However, the increased *MALAT1* expression level were shown to be associated with the better OS (*P* = 0.028) (Fig. 3d), in comparison with that when low expression of these lncRNAs was detected. Furthermore, by univariate analysis, we detected significant correlations between OS and *MALAT1* expression in HCC ≥ median (hazard ratio [HR], 0.58; 95% confidence interval (CI), 0.36 to 0.95; *P* = 0.03) as well as prothrombin time < 70% (HR, 2.03; 95% CI, 1.11 to 3.72; *P* = 0.02), B or C liver damage (HR, 2.09; 95% CI, 1.21 to 3.62; *P* = 0.01), multiple tumor (HR, 1.68; 95% CI, 1.00 to 2.81; *P* = 0.049), AFP ≥ 20 ng/mL (HR, 2.15; 95% CI, 1.35 to 3.45; *P* = 0.0 01) poorly differentiated HCC (HR, 2.29; 95% CI, 1.14 to 4.62; *P* = 0.02), serosal invasion (HR, 2.43; 95% CI, 1.44 to 4.11; *P* = 0.001), vascular invasion (HR, 2.39; 95% CI, 1.47 to 3.87; *P* = 0.0004), and positive surgical margin (HR, 1.94; 95% CI, 1.09 to 3.45; *P* = 0.02) (Table 2).

**Table 2:** Univariate analysis of overall survival.

Variables		HR	95% CI		
			Low	High	*P*
Age (years)	≥65 vs <65	1.53	0.96	2.45	0.08
Sex	Male vs female	1.18	0.62	2.24	0.62
Virus infection	HCV vs others	1.46	0.90	2.38	0.12
Albumin (g/dL)	<3.5 vs ≥3.5	1.66	0.98	2.82	0.06
PT (%)	<70 vs ≥70	2.03	1.11	3.72	0.02
ICG-R15 (%)	≥15 vs <15	1.75	0.95	3.23	0.07
Liver cirrhosis	(+) vs (−)	1.26	0.78	2.03	0.35
Child-Pugh classification	B vs A	1.64	0.71	3.79	0.25
Liver damage	B or C vs A	2.09	1.21	3.62	0.01
Tumor number	Multiple vs solitary	1.68	1.00	2.81	0.05
Tumor size (cm)	≥2 vs <2	2.40	0.96	5.96	0.06
AFP (ng/mL)	≥20 vs <20	2.15	1.35	3.45	0.001
Differentiation	Poor vs well/moderate	2.29	1.14	4.62	0.02
Growth form	Infiltrative vs expansive	1.66	0.94	2.95	0.08
Formation of capsule	(−) vs (+)	1.20	0.71	2.02	0.51
Infiltration to capsule	(+) vs (−)	1.02	0.64	1.63	0.93
Septal formation	(−) vs (+)	0.99	0.60	1.64	0.98
Serosal invasion	(+) vs (−)	2.43	1.44	4.11	0.001
Portal vein or hepatic vein invasion	(+) vs (−)	2.39	1.47	3.87	0.0004
Surgical margin	(+) vs (−)	1.94	1.09	3.45	0.02
Stage	III/IV vs I/II	1.50	0.94	2.41	0.09
*HULC* expression in HCC	≥High vs <Low	0.63	0.36	1.10	0.10
*MALAT1* expression in HCC	≥High vs < Low	0.58	0.36	0.95	0.03

HR; hazard ratio; CI, confidence interval; HCV, hepatitis C virus; PT, prothrombin time; ICG-R15, indocyanine green 15-min retention rate; AFP, alpha fetoprotein; HULC, Hepatocellular Carcinoma Up-Regulated Long Non-Coding RNA; HCC, hepatocellular carcinoma; MALAT1, Metastasis Associated Lung Adenocarcinoma Transcript 1.

**Figure 4:**
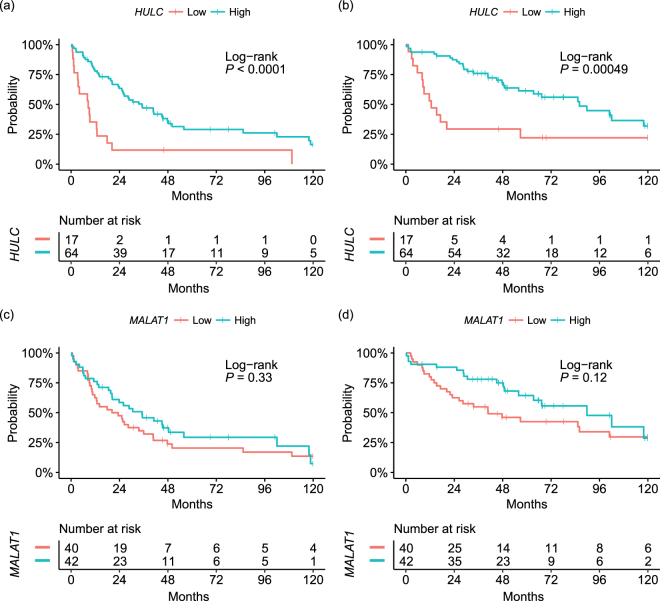
Survival analysis stratified by *HULC* and *MALAT1* expression levels in HCC tissues from HCV patients. (**a**) RFS analysis stratified by *HULC* expression. (**b**) OS analysis stratified by *HULC* expression. (**c**) RFS analysis stratified by *MALAT1* expression. (d) OS analysis stratified by *MALAT1* expression. HULC, Hepatocellular Carcinoma Up-Regulated Long Non-Coding RNA; MALAT1, Metastasis Associated Lung Adenocarcinoma Transcript 1; OS, overall survival; RFS, recurrence-free survival.

The cohort used in this study is composed of cases with HBV 38 (24%), HCV 89 (56%), HBV + HCV 3 (2%), and None-virus 28 (18%). This proportion is similar to those in the EU, US, and Japan, as reported previously^18^. Then, using only HCV cases (81 informative cases with RFS and 82 informative cases with OS), a prognostic analysis was performed. Interestingly, the result revealed that the RFS and OS value, as stratified by the expression level of *HULC* was significantly different (RFS: HR, 0.30; 95% CI, 0.17 to 0.55; *P* < 0.0001; OS: HR, 0.32; 95% CI, 0.17 to 0.63; *P* = 0.0005, Fig. 4a and b). However the RFS and OS value, as stratified by the expression level of *MALAT1* had the same tendency as seen in entire cohort without any significant difference (RFS: HR, 0.78; 95% CI, 0.48 to 1.3; *P* = 0.33, OS: HR, 0.62; 95% CI, 0.34 to 1.1; *P* = 0.12, Fig. 4c and d).

### *HULC* and *MALAT1* Correlation with HCC Prognosis in a Public Available Dataset

Finally, we confirmed the impact of *HULC* and *MALAT1* expression on HCC prognosis using TCGA dataset. Based on the normalized RNA-sequencing data, HCC cases were divided into two groups, according to the *HULC* and *MALAT1* expression levels in HCC tissues (HULC: < 0.45 and ≥ 0.45; MALAT1: < 9.35 and ≥ 9.35). As a result, the prognosis of HCC cases from TCGA stratified by *HULC* and *MALAT1* expression have the same tendency for HCC prognosis as seen in our collected 158 cases (Supplementary Figure S1).

## Discussion

With the development of whole genome and transcriptome sequencing, many lncRNAs were shown to be highly expressed in tumor tissue^19^. Gene expression profile of the surrounding normal liver tissue is important for HCC prognosis and the potential for recurrence^12^. We attempted to identify lncRNAs that may be used as prognostic biomarkers for HCC, by comparing their expression levels in the non-tumorous background liver tissue of HCC patients and non-tumorous and non-hepatitis liver tissue from the metastatic hepatic cancer patient^12^. In this way, we found five lncRNAs, shown to be highly expressed in CN, and the expression profiles obtained using a small-sized cohort confirmed that *HULC* and *MALAT1* show higher expression in the CN than SN samples. According to the cancer genome atlas (TCGA) dataset, *HULC* and *MALAT1* are highly expressed in the HCC tissues (*HULC*: 371 tumor *vs*. 50 normal, fold change 2.04; *MALAT1*: 373 tumor *vs*. 50 normal, fold change 1.72)^20^.

HULC was first described as a lncRNA with induced expression in HCC compared with the non-neoplastic tissue^21^. In osteosarcoma and pancreatic cancer, an increased expression of HULC in tumor tissue was shown to indicate poor prognosis^22,23^. MALAT1 is highly conserved and it was originally reported as a biomarker of metastasis in the early lung adenocarcinoma^24^. Functionally, HULC has not been completely elucidated, and a previous report showed that HULC can act as an oncogene in HCC through the deregulation of lipid metabolism through a signaling pathway involving miR-9, PPARA, and ACSL1^25^. MALAT1 acts as a proto-oncogene through the Wnt pathway activation and induction of the oncogenic splicing factor SRSF1^26^.

In this study, we showed that the expression of *HULC* was significantly higher in HCC samples, but the expression of *MALAT1* was not showed to be altered between CN and HCC. This may be caused by the presence of hepatitis in these cases. The underlying cause of hepatitis differed between the samples and in each study, the distribution of causes for hepatitis may vary. Furthermore, in breast cancer, the expression pattern of MALAT1 differs between transcript variants^23^, indicating that the transcript variant should be considered when determining the expression level of these lncRNAs in HCC^27,28^.

Unexpectedly, we did not observe the difference in lncRNA expression profiles between the background liver tissue and HCC tissue, and no correlation between *HULC* and *MALAT1* levels in CN and other background factors was determined. However, we demonstrated a significant difference between the expression of these two lncRNAs in HCC and other clinicopathological tumor-related factors, indicating that the decreased aggressiveness of HCC may be associated with the expression level of both *HULC* and *MALAT1*. Additionally, we demonstrated that the induced *HULC* and *MALAT1* expression in HCC correlates with a better prognosis. Few reports demonstrated that these two genes contribute to tumor development and recurrence^26,29^ and they are tumor indicators^30^. The preliminary *HULC* analysis with Gene Expression Omnibus (GEO) and *in silico* analysis showed that the higher expression of *HULC* is correlated with the better prognosis in HCC^31^. To the best of our knowledge, the relationship between these two lncRNAs and HCC prognosis, using clinical samples, has not been established previously. Our results indicate that these two lncRNAs may act as oncogenes, but they do not accelerate the progression of HCC.

This study is limited due to the use of a single-center cohort, without the possibility of the analysis of an independent cohort. In order to confirm these results further, they should be validated using an independent HCC cohort.

In conclusion, our findings suggest that the increased expression of *HULC* and *MALAT1* in HCC tissue may represent a good prognostic biomarker for curatively resected HCC. Therefore, in combination with other tumor prognostic factors, the determination of the expression levels of these lncRNAs may lead to a more accurate prediction of HCC prognosis.

## Electronic supplementary material

Supplementary material
