# Supplementary material for: Prognostic Value of Long Non-Coding RNA HULC and MALAT1 Following the Curative Resection of Hepatocellular Carcinoma
Source: Sci Rep. 2017 Nov 23;7:16142. doi: 10.1038/s41598-017-16260-1 (PMC5700934; doi:10.1038/s41598-017-16260-1)

Prognostic Value of Long Non-Coding RNA HULC and MALAT1 Following the Curative Resection of Hepatocellular Carcinoma

Authors:

Fuminori Sonohara, MD, PhD[1,2]; Yoshikuni Inokawa, MD, PhD[1,2]; Masamichi Hayashi, MD, PhD[1]; Suguru Yamada, MD, PhD[1]; Hiroyuki Sugimoto, MD, PhD[1]; Tsutomu Fujii, MD, PhD[1]; Yasuhiro Kodera, MD, PhD[1]; Shuji Nomoto, MD, PhD*[1,2]

Affiliations:

1. Department of Gastroenterological Surgery, Nagoya University Graduate School of Medicine, Nagoya, Japan
2. Department of Surgery, Aichi-Gakuin University School of Dentistry, Nagoya, Japan

* Corresponding author

Contents:

Supplementary Table S1.

lncRNA more highly expressed in CN than SN derived from expression microarray.

Supplementary Table S2.

Clinical features stratified by *HULC* expression.

Supplementary Table S3.

Clinical features stratified by *MALAT1* expression.

Supplementary Figure S1.

Survival analysis stratified by *HULC* and *MALAT1* expression levels in HCC tissues from TCGA dataset. (a) RFS analysis stratified by *HULC* expression. (b) OS analysis stratified by *HULC* expression. (c) RFS analysis stratified by *MALAT1* expression. (d) OS analysis stratified by *MALAT1* expression. HULC, Hepatocellular Carcinoma Up-Regulated Long Non-Coding RNA; MALAT1, Metastasis Associated Lung Adenocarcinoma Transcript 1; OS, overall survival; RFS, recurrence free survival.

Supplementary Table S1. lncRNA more highly expressed in CN than SN derived from expression microarray.

GeneSymbol	RefSeqAccession	Fold change CN vs SN	Regulation CN vs SN
UCA1	NR_015379	7.981	Up
HULC	NR_004855	3.492	Up
MALAT1	NR_002819	1.852	Up
GAS5	NR_002578	1.286	Up
TUG1	NR_002323	1.271	Up

lncRNA, long non-coding RNA; CN, corresponding normal; SN, super normal; UCA1, Urothelial Cancer Associated 1; HULC, Hepatocellular Carcinoma Up-Regulated Long Non-Coding RNA; MALAT1, Metastasis Associated Lung Adenocarcinoma Transcript 1; GAS5, Growth Arrest Specific 5; TUG1, Taurine Up-Regulated 1.

Supplementary Table S2. Clinical features stratified by *HULC* expression.

Variables		*HULC* expression		*P*
		Low	High	
Age (years)	< 65	14	18	0.87
	≥ 65	54	60	
Sex	Female	6	26	0.99
	Male	19	95	
Virus infection	Others	14	18	1.00
	HCV	48	66	
Albumin (g/dL)	≥ 3.5	27	5	0.58
	< 3.5	88	25	
PT (%)	≥ 70	26	6	0.37
	< 70	100	13	
ICG-R15 (%)	< 15	14	8	0.33
	≥ 15	63	19	
Liver cirrhosis	Negative	20	12	1.00
	Positive	69	42	
Child-Pugh classification	A	30	2	1.00
	B	106	7	
Liver damage	A	24	7	0.68
	B or C	91	19	
Tumor number	Solitary	22	10	0.23
	Multiple	92	22	
Tumor size (cm)	< 2	6	24	0.58
	≥ 2	17	92	
AFP (ng/mL)	< 20	12	18	0.09
	≥ 20	68	46	
Differentiation	Well or moderate	27	4	0.29
	Poorly	104	8	
Growth form	Expansive	25	7	0.46
	Infiltrative	95	16	
Formation of capsule	Positive	11	21	0.78
	Negative	34	80	
Infiltration to capsule	Negative	13	18	0.87
	Positive	52	62	
Septal formation	Positive	12	18	0.33
	Negative	32	80	
Serosal invasion	Negative	23	6	0.94
	Positive	81	25	
Portal vein or hepatic vein invasion	Negative	19	13	0.08
	Positive	84	25	
Surgical margin	Negative	22	8	0.23
	Positive	90	16	
Stage	< III	16	16	0.12
	≥ III	75	37	

HULC, Hepatocellular Carcinoma Up-Regulated Long Non-Coding RNA; HCV, hepatitis C virus; PT, prothrombin time; ICG-R15, indocyanine green 15-min retention rate; AFP, alpha fetoprotein.

Supplementary Table S3. Clinical features stratified by *MALAT1* expression.

Variables		*MALAT1* expression		*P*
		Low	High	
Age (years)	< 65	34	39	1.00
	≥ 65	35	39	
Sex	Female	11	62	0.69
	Male	14	60	
Virus infection	Others	31	42	1.00
	HCV	31	43	
Albumin (g/dL)	≥ 3.5	58	15	1.00
	< 3.5	58	15	
PT (%)	≥ 70	60	13	0.14
	< 70	67	6	
ICG-R15 (%)	< 15	41	12	0.61
	≥ 15	37	15	
Liver cirrhosis	Negative	43	29	0.61
	Positive	47	25	
Child-Pugh classification	A	69	4	1.00
	B	68	5	
Liver damage	A	58	13	1.00
	B or C	58	13	
Tumor number	Solitary	57	16	1.00
	Multiple	57	17	
Tumor size (cm)	< 2	6	63	0.02
	≥ 2	17	53	
AFP (ng/mL)	< 20	32	39	0.02
	≥ 20	49	25	
Differentiation	Well or moderate	65	6	1.00
	Poorly	66	6	
Growth form	Expansive	60	12	1.00
	Infiltrative	61	11	
Formation of capsule	Positive	17	56	0.08
	Negative	28	46	
Infiltration to capsule	Negative	30	42	0.50
	Positive	36	38	
Septal formation	Positive	21	49	0.85
	Negative	24	49	
Serosal invasion	Negative	53	15	1.00
	Positive	52	16	
Portal vein or hepatic vein invasion	Negative	46	24	0.11
	Positive	57	15	
Surgical margin	Negative	56	13	0.85
	Positive	57	11	
Stage	< III	41	32	0.14
	≥ III	50	22	

MALAT1, Metastasis Associated Lung Adenocarcinoma Transcript 1; HCV, hepatitis C virus; PT, prothrombin time; ICG-R15, indocyanine green 15-min retention rate; AFP, alpha fetoprotein.

Supplementary Figure S1

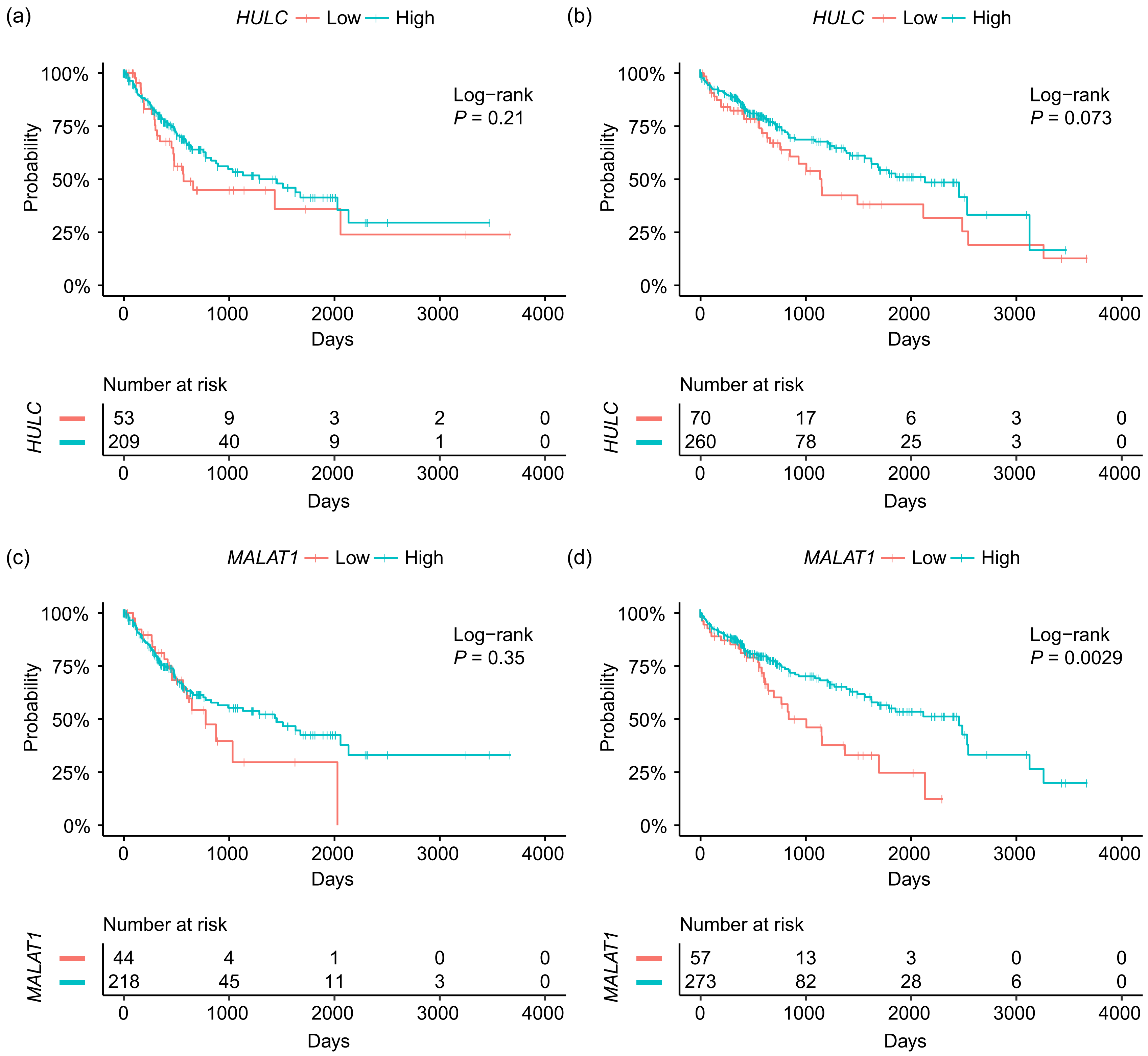

Supplement: Supplementary file 1 — Supplementary material [file 41598_2017_16260_MOESM1_ESM.pdf]
